# Defining the combined stress response in wild *Arachis*

**DOI:** 10.1038/s41598-021-90607-7

**Published:** 2021-05-27

**Authors:** Ana Paula Zotta Mota, Ana Cristina Miranda Brasileiro, Bruna Vidigal, Thais Nicolini Oliveira, Andressa da Cunha Quintana Martins, Mario Alfredo de Passos Saraiva, Ana Claudia Guerra de Araújo, Roberto C. Togawa, Maria Fatima Grossi-de-Sá, Patricia Messenberg Guimaraes

**Affiliations:** 1 EMBRAPA Recursos Geneticos e Biotecnologia, Brasilia, DF Brazil; 2 Universidade Federal do Rio Grande do Sul, Porto Alegre, RS Brazil; 3 National Institute of Science and Technology-INCT PlantStress Biotech-EMBRAPA, Brasilia, Brazil; 4 Universidade Católica de Brasília (UCB)-Genomic Sciences and Biotechnology, Brasilia, DF Brazil; 5 Present Address: CIRAD, UMR AGAP, 34398 Montpellier, France; 6 Present Address: AGAP, Univ Montpellier, CIRAD, INRA, Montpellier SupAgro, Montpellier, France

**Keywords:** Abiotic, Biotic, Transcriptomics, Molecular engineering in plants

## Abstract

Nematodes and drought are major constraints in tropical agriculture and often occur simultaneously. Plant responses to these stresses are complex and require crosstalk between biotic and abiotic signaling pathways. In this study, we explored the transcriptome data of wild *Arachis* species subjected to drought (A-metaDEG) and the root-knot nematode *Meloidogyne arenaria* (B-metaDEG) via meta-analysis, to identify core-stress responsive genes to each individual and concurrent stresses in these species. Transcriptome analysis of a nematode/drought bioassay (cross-stress) showed that the set of stress responsive DEGs to concurrent stress is distinct from those resulting from overlapping A- and B-metaDEGs, indicating a specialized and unique response to combined stresses in wild *Arachis*. Whilst individual biotic and abiotic stresses elicit hormone-responsive genes, most notably in the jasmonic and abscisic acid pathways, combined stresses seem to trigger mainly the ethylene hormone pathway. The overexpression of a cross-stress tolerance candidate gene identified here, an endochitinase-encoding gene (*AsECHI*) from *Arachis stenosperma*, reduced up to 30% of *M. incognita* infection and increased post-drought recovery in *Arabidopsis* plants submitted to both stresses. The elucidation of the network of cross-stress responsive genes in *Arachis* contributes to better understanding the complex regulation of biotic and abiotic responses in plants facilitating more adequate crop breeding for combined stress tolerance.

## Introduction

Plants are constantly exposed to concurrent environmental stresses, such as drought and diseases which cause significant yield losses^1,2^. In addition, the foreseen impacts of climate change on crop productivity, including water shortage and disease emergence, stand a great challenge for global food security, propelling the need for the development of more adapted cultivars^3^. Over the course of evolution, plants developed complex networks to overcome stresses that rely on their ability to perceive external signals and activate an appropriate spectrum of molecular responses in a timely manner^4,5^. This include different signaling pathways mobilized to respond to abiotic and biotic stresses^6–8^, that in the case of simultaneous occurrence, lead to synergism or the prioritization of one stress response over another, often causing a decrease in plant tolerance to other hazards and losses in productivity^8^. The elucidation of key elements in plant multi-layered defense to simultaneous stresses provides new opportunities for enhancing plant resistance and can largely contribute to the improvement of crop yield^9–11^.

To date, few studies on plant responses to simultaneous abiotic and biotic stresses have been conducted^12–16^, with the majority reporting increased resistance to fungi and abiotic stresses due to enhanced levels of annexins^17^, osmotin-like proteins^18^, polyamines^19,20^ or transcription factors^21–23^. Nonetheless, no studies on concurrent improvement for nematode and abiotic resistances have yet been reported.

The role of phytohormones in activating the defense responses against biotic and abiotic stresses is well- known, with salicylic acid (SA) mediating responses mostly to biotrophic pathogens, jasmonic acid (JA) and ethylene (ET) to chewing insects and necrotrophic pathogens, and abscisic acid (ABA) to abiotic stresses^24–26^. In addition, SA and JA/ET defense pathways often act as mutually antagonistic^27,28^, with ABA appearing as a central modulator of the regulatory crosstalk between these phytohormones, directly impacting SA and JA biosynthesis^13,29^. The crosstalk between ABA/JA-ET^30^, ABA/SA^31^ and JA/SA^32^, that often causes antagonistic effects, is important to guarantee that those hormone cascades with low or no significant effects on the pathogen control are suppressed to avoid the waste of valuable physiological resources^33^. Nonetheless, a universal phytohormonal interplay does not apply to all cases, with the outcome of ABA and SA-JA/ET interaction being largely dependent upon the pathosystem, plant developmental stage, as well as on the timing of infection^34,35^.

Peanut wild relatives (*Arachis* spp.) have evolved and adapted to a wide range of environments and harbor high levels of resistance to many pathogens and abiotic constraints^36–39^, constituting useful sources of resistance alleles against these stresses. Previous studies showed that *Arachis stenosperma* is highly resistant to the Root-knot nematode (RKN) *Meloidogyne arenaria,* triggering the Hypersensitive Response (HR) at the nematode feeding site^40,41^, whilst *A. duranensis* is more tolerant to water deficit by displaying a more conservative plant transpiration behavior^42^. While *A. stenosperma* HR response against *M. arenaria* has been linked to the activation of the JA pathway, the ABA-independent signaling pathway seems to be the main activator of the adaptive physiological changes in the drought-tolerant *A. duranensis*^39,43^.

Nonetheless, the core stress-responsive regulatory mechanisms and agents involved in wild *Arachis* response to concurrent abiotic and biotic stresses have not yet been elucidated.

The development of meta-analysis methods associated with low sequencing costs (RNA-Seq), has enabled the comparison among transcriptomes from the most diverse conditions^44^. However, to date, few studies have exploited data from biotic and abiotic meta-analysis^11^ and none of these studies compared the genes obtained from this analysis to a de facto combined stress transcriptome.

In the present study, we investigated the transcriptional dynamics elicited by RKN infection in combination with drought, via the meta-analysis of our previously produced wild *Arachis* RNA-Seq data, and evaluated the use of this approach as a predictive tool for the discovery of genes responsive to combined stresses. We also evaluated the effect of the endochitinase (*AsECHI*) overexpression, a cross-stress gene identified here, on the improvement of *Arabidopsis thaliana* plants’ tolerance to combined nematode and drought stresses. Understanding the complex regulation of biotic and abiotic responses and their interactions is crucial to avoid trade-off effects that limit plant growth and yield and will facilitate a more efficient crop breeding for combined stress tolerance.

## Results

### Meta-analysis of *Arachis* transcriptome reveals core stress-responsive genes

**Figure 1:**
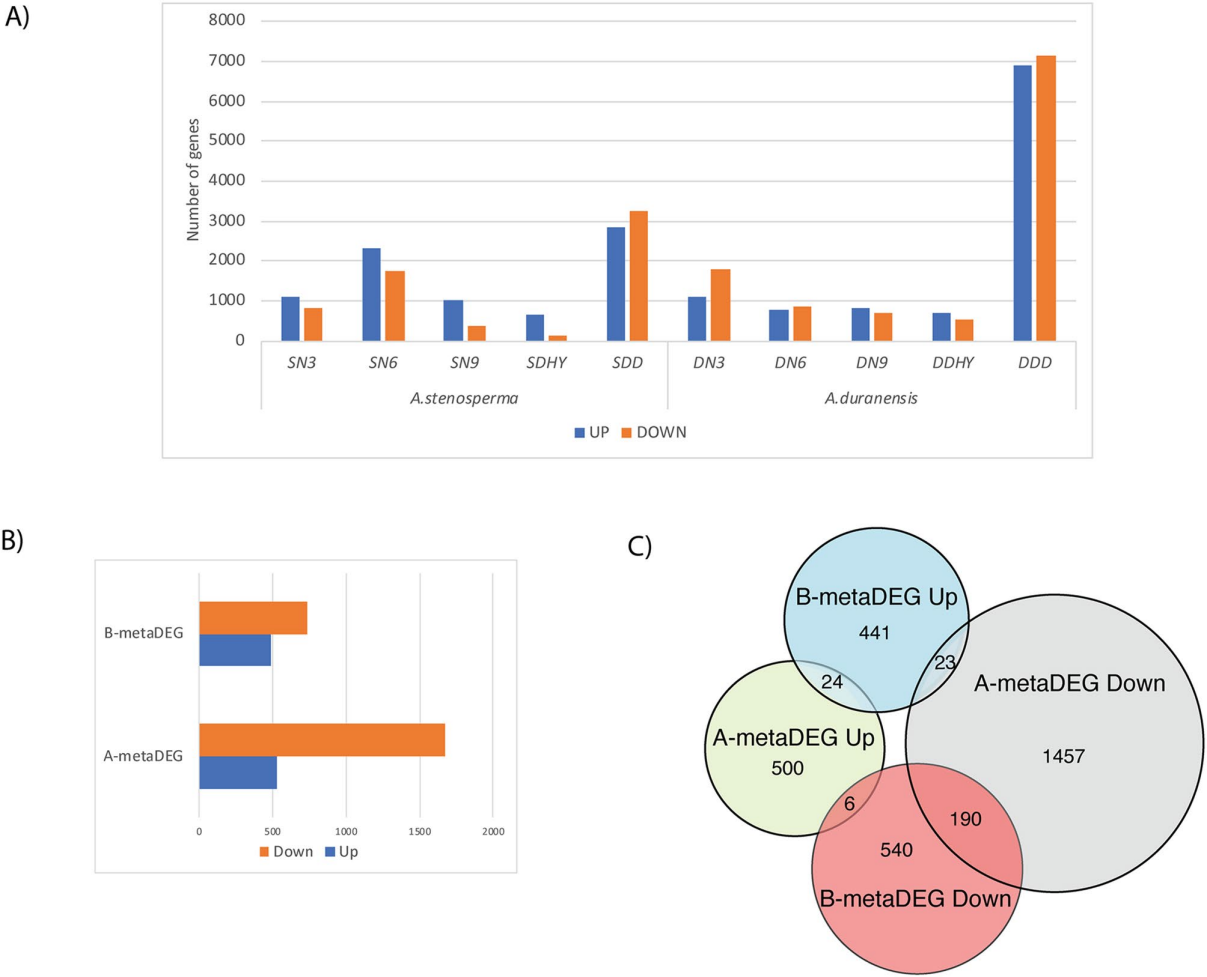
*Arachis* spp transcriptome libraries used in the meta-analysis: **(A)** Number of DEGs up- and downregulated in *A. stenosperma* and *A. duranensis* plants submitted to biotic and abiotic stress. **(B)** Number and expression trend of metaDEGs found in biotic and abiotic stress categories. **(C)** Euler diagram showing metaDEGs distribution according to stress category and their expression trend. *A. stenosperma* inoculated with nematode at 3DAI, 6DAI and 9DAI (SN3, SN6, SN9), *A. stenosperma* submitted to dehydration (SDHY) and dry-down (SDD). *A. duranensis* inoculated with nematode at 3DAI, 6DAI and 9DAI (DN3, DN6, DN9), *A. duranensis* submitted to dehydration (DDHY) and dry-down (DDD).

Our previously produced RNA-Seq data consisting of 16 cDNA libraries (Supplementary Table S1) from two wild *Arachis* species under biotic and abiotic stresses were explored to identify unique and commonly stress-regulated genes. The overall expression trend and number of DEGs varied for each species/treatment combination, with the dry-down treatment (abiotic stress) prompting the highest number of responsive DEGs in both species (Fig. 1A). The drought-tolerant *A. duranensis* showed the greatest number of DEGs in response to abiotic stress (*A. duranensis* dehydration (DDHY) + *A. duranensis* dry-down (DDD)), whilst the RKN-resistant *A. stenosperma* displayed the greatest number of DEGs in response to biotic stress (Fig. 1A), coinciding with the occurrence of HR to nematode inoculation at 6 DAI (SN6)^40^. As previously observed^39^, similar distribution between up and downregulated DEGs was identified in *A. duranensis* during the abiotic stress, whilst for *A. stenosperma* more DEGs were upregulated under nematode infection^43^ (Fig. 1A).

For meta-analysis, we analyzed separately the DEGs from abiotic (A) and biotic (B) libraries. Genes with the same expression trend among the abiotic and/or biotic were considered as A- and B-metaDEGs. By following these criteria, we assume that a better representation of the overall expression trend of the stress-responsive genes during the time of the stress imposition was obtained^11^.

Overall, we found 2,200 DEGs responsive to abiotic stress in the meta-analysis (A-metaDEGs) with the majority downregulated (1670; 75.9%) and 530 upregulated (24.09%) (Fig. 1B,C; Supplementary Table S2) in both species (*A. duranensis* and *A. stenosperma*). A total of 1,224 B-metaDEGs were identified, being 736 DEGs (60.08%) downregulated and 488 upregulated (39.83%) in both species (Fig. 1B,C; Supplementary Table S3).

In sum, only 7% (243) of all A- and B-metaDEGs were common between biotic and abiotic stresses, with the great majority (213) showing downregulation (Fig. 1C). This set of 243 metaDEGs, hereby called as wild *Arachis* core-metaDEGs, were genes responsive to stress regardless of plant genotype, type or timing of the stress imposed, constituting a valuable tool for better understanding the nature of stress resistance/tolerance in the genus, and to be further exploited as novel wild alleles involved in adaptability to stressful environments.

### De facto combined stress analysis

To investigate the prediction power of the meta-analysis, we conducted a de facto combined stress assay and produced a new set of *A. stenosperma* transcriptome libraries submitted to drought, nematode inoculation and combined stresses (cross-stress). In total, we found 3,345 DEGs in the cross-stress library (crossDEGs) (Supplementary Table S4), 6,102 DEGs in the exclusive drought and 1,251 DEGs in the nematode infection libraries (Supplementary Table S5).

**Figure 2:**
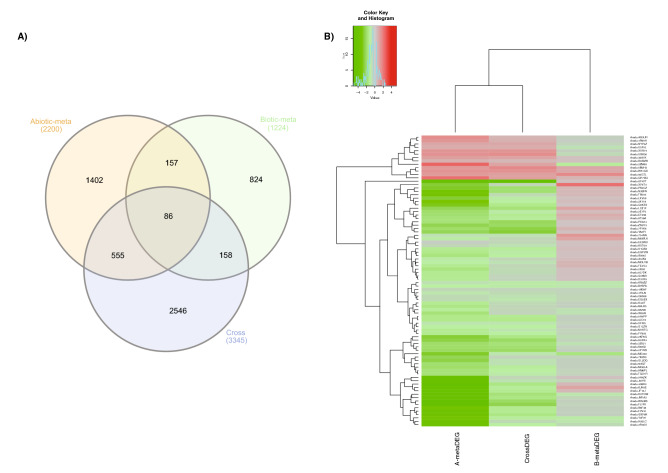
Comparison between predicted genes from (A- and B-metaDEGs) and observed de facto cross-stress DEGs (crossDEGs). **(A)** Venn diagram of A-metaDEGs, B-metaDEGs and crossDEGs; **(B)** expression profile of the 86 common DEGs in response to de facto cross-stress (crossDEGs) and to all abiotic (A-metaDEGs) and biotic (B-metaDEGs) treatments using gplots. The color key represents the values of log2FC.

The comparison between the 3,345 de facto crossDEGs and the 3,424 A- and B-metaDEGs intersection, identified 86 DEGs in common (Fig. 2A), representing 35.4% of the 243 core-metaDEGs (overlapping A- and B-metaDEGs). This de facto analysis therefore corroborated the meta-analysis on that biotic and abiotic stresses not only prompt different sets of genes in *Arachis* but also trigger distinct molecular responses when applied individually or in combination. This unique and complex transcript plant response to stress combinations has also been observed for other species^45^. In addition, this analysis enabled the identification of a set of genes (86) modulated in response to combined stress and validated by the meta-analysis, which are highly valuable for genetic breeding and biotechnological purposes.

Interestingly, the expression trend of these 86 common genes showed to be more comparable to A-metaDEGs than B-metaDEGs, suggesting a dominance of the drought stress response over the biotic stress (Fig. 2B; Supplementary Table S6).

### Functional annotation of *Arachis* stress responsive genes

**Figure 3:**
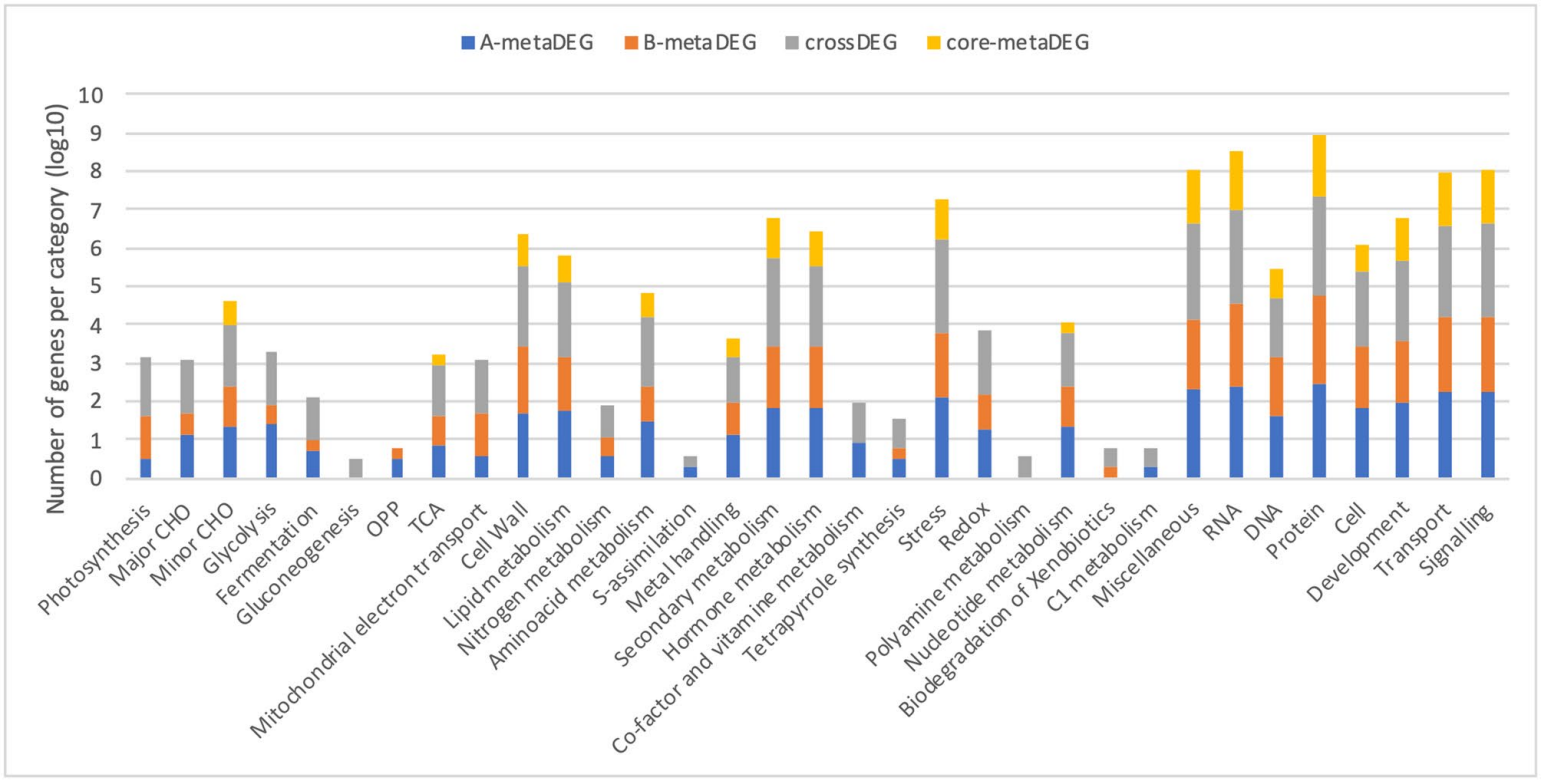
Number of genes annotated in each Mercator categories for the A-metaDEG, B-metaDEG, crossDEG and core-metaDEG. The number of genes is represented in log10.

Over 70% of the 6,769 *Arachis* DEGs identified in the meta- (3,424) and de facto (3,345) analyses could be assigned to 33 different functional categories (Fig. 3). From these, Protein and RNA categories (including Transcription Factors; TFs) were the most represented, followed by categories involved in plant basic metabolism, such as Secondary Metabolism and Transport. In total, 416 DEGs were assigned to the Stress Category, which comprised pathogenesis-related proteins, NLRs, chaperones, and HSP (heat-shock proteins) (Fig. 3).

A high number of B-metaDEGs were classified in the cell wall and lipid metabolism which could be related to the formation of the first barrier of plant defense against the nematode infection, whilst the A-metaDEGs in the hormone and secondary metabolism categories might be involved in triggering the drought response.

### The whole is not equal to the sum of the parts

Overall, low predictability of the meta-analysis to identify de facto cross-stress responsive genes was observed, with 19.2% (641) genes forecast as A-metaDEGs, 7.3% (244) as B-metaDEGs and only 2.6% (86) as core-metaDEGs (Fig. 2A). Also, the comparison between the de facto crossDEGs and core-metaDEGs (Fig. 3), showed that genes belonging to some categories directly or indirectly involved in plant resistance, such as Redox, Photosynthesis and Mitochondrial electron transport, were missing in the core-metaDEGs^46–48^, suggesting that novel defense responsive genes and pathways are triggered in response to simultaneous stresses which are not activated when the corresponding individual stress is applied.

**Figure 4:**
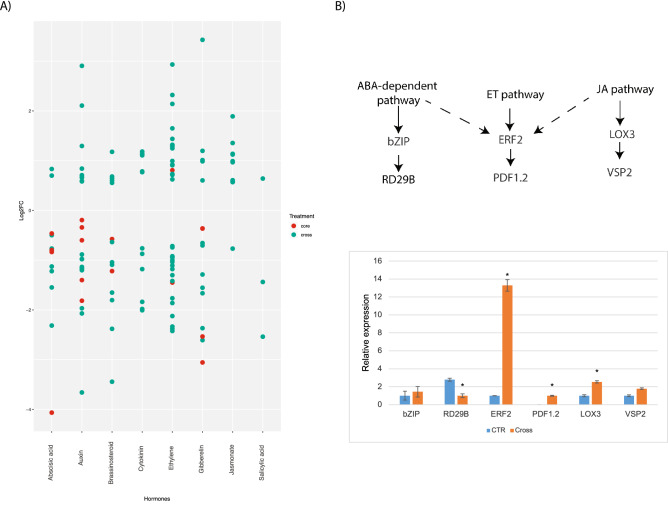
**(A)** Dot-plot of the expression patterns of *Arachis* DEGs involved in hormone pathways: red-core-metaDEGs; green-crossDEGs. Each dot represents the relative expression value (log2FC) of a DEG in the y-axis; **(B)** qRT-PCR: relative quantification of mRNA levels of marker genes in the ABA, JA and ET pathways in *A. stenosperma* roots submitted to cross-stress treatment relative to non-treated control samples. Values are the mean standard deviation of three biological replicates and * means significant differences (p < 0.05). *NAS* not amplified sample.

Considering the importance of plant hormones in the signaling and activation of defense responses against both abiotic and biotic stresses, we depicted the expression trend of the predicted stress-responsive genes (core-metaDEGs and crossDEGs) into the main hormone pathways involved in plant defense (Fig. 4A). Over 124 crossDEGs were found to be related to ABA, auxin, brassinosteroids, cytokinin, ET, gibberellin, JA and SA pathways (Fig. 4A). From these, cytokinin, JA and SA hormone families were those containing the majority of the genes, albeit with low expression (Fig. 4A). On the other hand, only nine core-metaDEGs were found in defense hormone categories (Fig. 4A), with the majority related to hormones involved in plant development, such as auxin and gibberellin.

In previous transcriptome studies in wild *Arachis*, we demonstrated that the majority of the drought responsive genes involved in hormonal signaling were related to the ABA pathway^39^, while most genes responsive to RKN infection belonged to the JA pathway^43,49^. Nonetheless, the analysis of the de facto crossDEGs showed that another important hormone alternative defense pathway, the ET pathway, was more activated, with 32 genes modulated and classified as crossDEGs, whilst the JA and ABA pathways showed only 11 and eight crossDEGs, respectively. This indicates a shift on the main responsive hormone pathways normally triggered during each of these stresses alone, (e.g.) ABA for abiotic and JA for biotic stresses (Fig. 4A).

It has been shown in Arabidopsis that ABA interferes with the signaling pathways regulating the JA-ethylene defense gene expression through the ethylene responsive factor (ERF), which integrates signals from both JA and ethylene pathways^50^. This negative regulation of defense gene expression by ABA and the antagonistic effect of ethylene on ABA signaling has been demonstrated in different species by the modulation of defense genes expression such as PDF1.2, VSP2 or the ABA responsive RD29^51–53^.

In this study, the qRT-PCR expression analysis of five *A. stenosperma* marker genes involved in ET, JA and ABA pathways corroborated this assumption. The expression of ERF2, a regulator of ET signaling, was strongly induced, while PDF1.2, a downstream ET‐responsive gene, was exclusively detected in the cross-stress treatment (Fig. 4B). Likewise, despite marker genes for the JA (LOX3 and VSP2) and ABA (bZIP and RD29B) pathways being also triggered during the multiple stress imposition, they exhibited a more discrete modulation and were even slightly downregulated, as for the RD29B gene (Fig. 4B).

Therefore, the activation of an alternative ET pathway, together with the mobilization of genes involved in the ABA and JA pathways, and the plausible *cross-talk* of these hormones signaling, appears as one of the fundamental components of the wild *Arachis* tolerance mechanism against simultaneous stresses.

### Plant functional validation of a crossDEG candidate (AsECHI)

**Figure 5:**
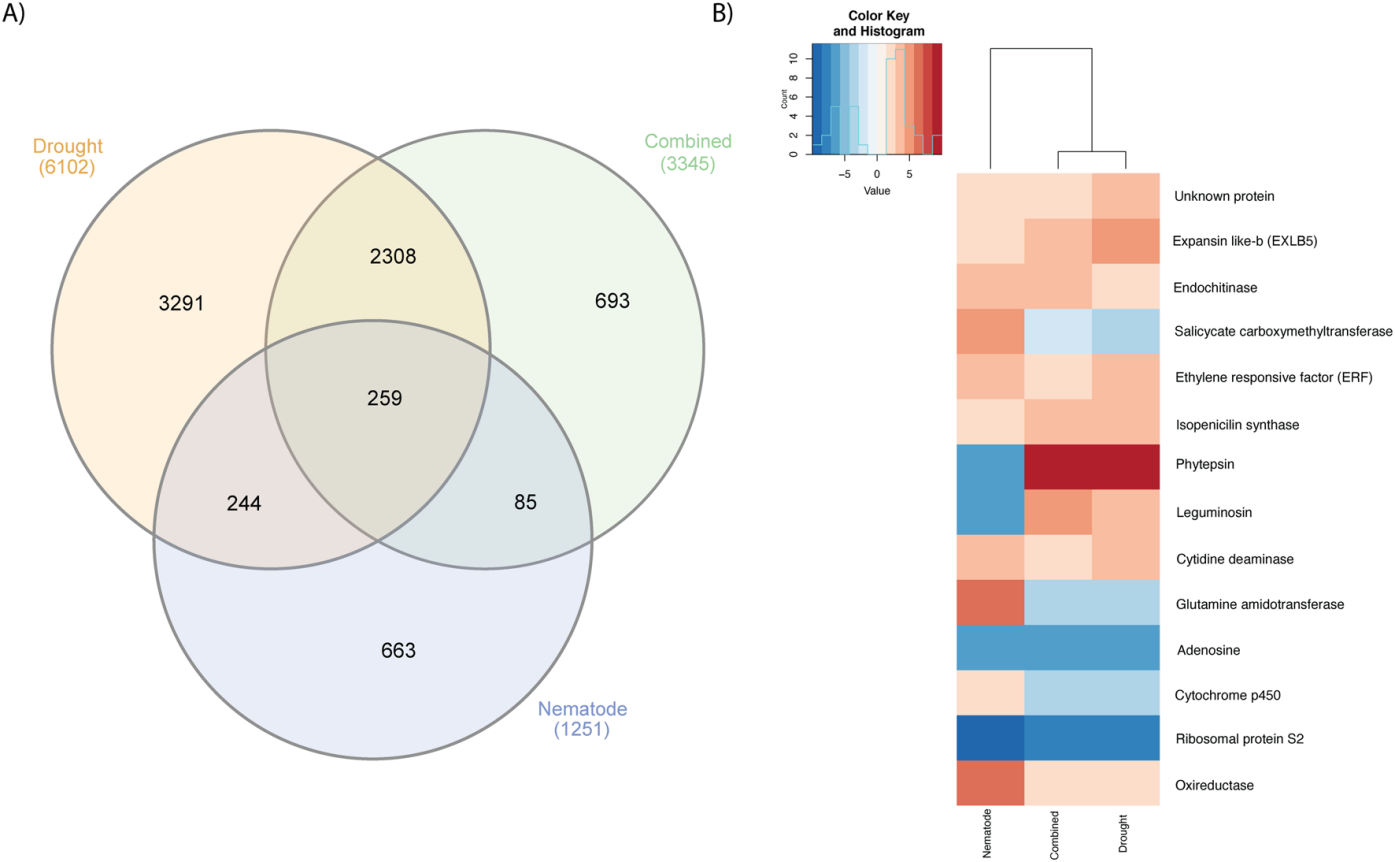
*A. stenosperma* DEGs from de facto transcriptome in response to biotic (nematode) and abiotic (drought) treatments and their combination (crossDEGs). **(A)** Venn diagram of de facto DEGs from the nematode, drought and combined treatments. **(B)** Expression values of 14 de facto DEGs with the highest expression values to all treatments heatmap generated by gplots.

In order to functionally validate candidate genes for multiple stress tolerance in *A. stenosperma*, we explored the 259 DEGs from de facto transcriptome analysis that were commonly responsive to the three stress treatments (drought, nematode and cross-stress) (Fig. 5A). Among these crossDEGs that encompassed transcription factors, cell-wall proteins, metal and ion transferases proteases, we analyzed the expression profile of the 14 de facto crossDEGs that showed the highest expression in response to these combined and isolated stresses (Fig. 5B; Supplementary Table S4). One of them (Aradu.4196P), encoding an endochitinase (ECHI), drew attention as it showed a strong positive regulation in roots under all treatments (Fig. 5B): drought (2.53-fold), nematode infection (3.96 fold) and combined stress (3.54 fold). In addition, this endochitinase was previously identified as a differentially expressed protein (DAP) induced by drought in our previous proteomic surveys in *A. duranensis*^54^, therefore being selected as our first candidate for functional analysis *in planta.*

The 795 bp consensus coding sequence of ECHI from *A. stenosperma* (*AsECHI*), was successfully cloned in the pPZP_BAR binary vector and used for *A. thaliana* transformation. The overexpression of *AsECHI* transgene was confirmed in four overexpressing (OE) lines (OE1, OE2, OE6 and OE7) at T2 generation by qRT-PCR analysis, with expression levels varying according to the transgenic line (Supplementary Figure S1). Plants from these four OE lines were submitted to drought and *M. incognita* inoculation independent assays, and the two best performing OE lines (OE1 and OE2) were further submitted to the combined stress experiment.

### Drought stress response and nematode infection in AsECHI-OE lines

Leaves of four *A. thaliana AsECHI*-OE lines submitted to dry-down treatment displayed a slightly curled phenotype at the 9th day of drought imposition, while WT plants were severely affected (Supplementary Figure S2). Increased RWC values were also observed in OE lines submitted to dry-down treatment when compared to WT, with OE2 and OE7 lines displaying significant differences of 34 and 20%, respectively (Supplementary Figure S2a). The opposite behavior was observed for EL values that were reduced during drought stress in OE lines when compared to the WT plants, with OE2, OE6 and OE7 lines displaying significant differences of 28, 29 and 24%, respectively (Supplementary Figure S2a). Under well-irrigated control treatment, no significant differences between OE lines and WT plants were observed for both RWC and EL measurements. The phenotype of transgenic plants in response to drought imposition, together with good indexes of water loss control (higher RWC values) and membrane stability (lower EL values), strongly indicate that *AsECHI* overexpression improved plant tolerance to low water availability in soil. The OE2 line drew particular attention as it showed the highest differences in comparison with WT plants for both RWC and EL values (Supplementary Figure S2a) and was therefore selected as the best line for further combined stress assay.

The same four *A. thaliana AsECHI*-OE lines were inoculated with 1000 J2 *M. incognita*. At 60 DAI, the nematode infection was confirmed through root galling and the presence of females in the inoculated plants (Supplementary Figure S2b). Roots from OE1, OE2 and OE7 lines showed a lower number of galls and 50, 37 and 40% of reduction in the number of females per gram of roots, respectively, in comparison to WT plants (Supplementary Figure S2). The OE1 had the greatest reduction in females per gram of root, and therefore was selected for further combined stress assay.

### Cross-stress response in AsECHI-OE lines

**Figure 6:**
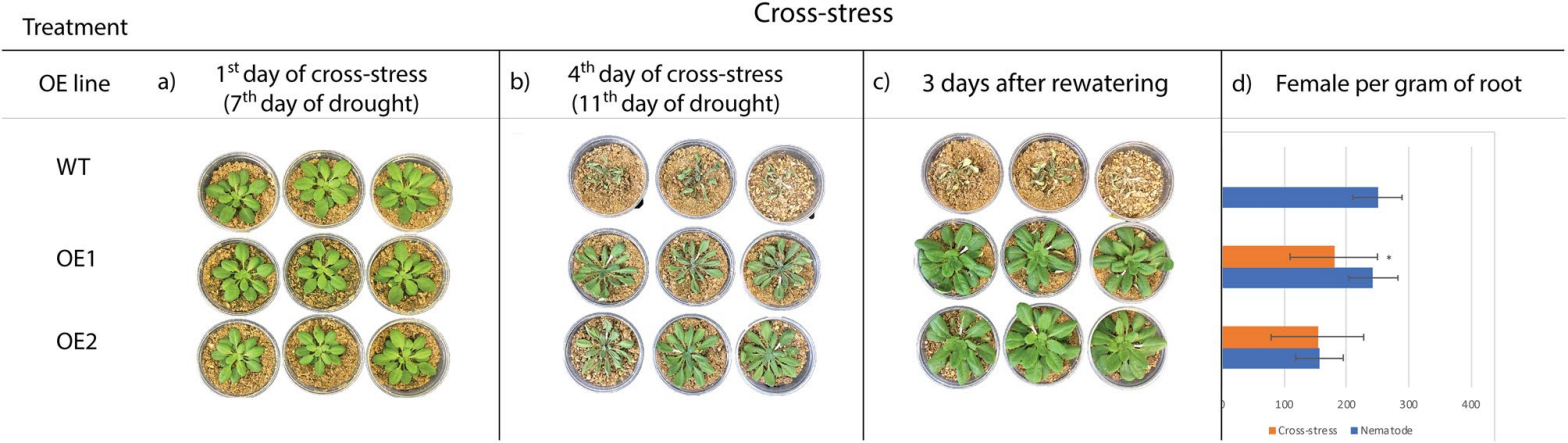
Cross-stress assay in *A. thaliana* OE1 and 2 lines and WT plants. **(a)** Plants at the 1st day of cross-stress treatment; **(b)** plants at the 4th day of cross-stress treatment; **(c)** plants at the 3rd day after rewatering; **(d)** female per gram of roots of plants submitted to the cross-stress and individual nematode treatments. Values are mean of 10 individuals and significant differences between nematode and cross-stress plants are marked with an asterisk.

The OE1 and OE2 lines selected above and WT plants were submitted to cross-stress treatment, in which plants submitted to water withdrawal for seven days were inoculated with 1000 J2 *M. incognita*. Well-irrigated and *M. incognita* inoculated plants were kept as non-treated and nematode infection controls, respectively. In accordance with the previous drought stress assay (Supplementary Figure S2a), at the 11th day of water withdrawal, leaves of the OE lines began to display water deficit symptoms (Fig. 6a,b) and were rewatered to 70% FC. Three days after rewatering, plants from both OE1 and OE2 lines fully recovered their well-irrigated phenotype, while WT plants seemed to reach permanent wilting point (Fig. 6c). Plants from the combined stress treatment were maintained under well-irrigated conditions until 60 DAI in order to enable the completion of the nematode cycle.

Nematode infection evaluation at 60 DAI showed that both OE lines displayed a reduction in the number of females per gram of root in relation to the control, but only OE1 was statistically significant (40.3%) (Fig. 6d). It is worth noticing that, as no WT plants survived to combined stress treatment, the nematode infection of OE lines was compared to the respective OE line inoculated solely with *M. incognita* (Fig. 6d).

As previously observed in the individual nematode inoculation assay, the overexpression of *AsECHI* contributed to the reduction of galling formation and female numbers in the transgenic roots under combined stress. In addition, the combination of drought imposition with nematode inoculation did not seem to affect the RKN resistance of the OE lines (Supplementary Figure S2; Fig. 6b).

Together, these results suggest that the *AsECHI* overexpression improved tolerance of transgenic plants to combined drought and RKN stresses, indicating that this gene could have an important role in the resistance of wild *Arachis* to multiple and combined stresses. It also reveals *AsECHI* as a promising candidate gene for biotechnological purposes for the improvement of plant tolerance against multiple abiotic and biotic stresses.

## Discussion

Due to climate change, a wider range of agricultural areas will be subjected to multiple stresses, including water scarcity and pathogens with expanded host ranges and increased virulence^55^. Recent studies have shown that plants tend to exhibit tailored physiological and molecular responses that occur only when they are exposed to simultaneous stresses^8,15,56^. In this light, a broader understanding of the crosstalk between hormone signaling pathways that have an essential role in plant responses to abiotic and biotic stresses is central for the development of more adapted and productive cultivars in the near future.

In this study, we explored our previously produced wild *Arachis* transcriptome data comprising 16 RNA-Seq libraries of plants submitted to drought and RKN infection by meta-analysis, in order to identify central genes putatively involved in plant defense against abiotic (A-metaDEGs), biotic (B-metaDEGs) and the combination of both stresses (core-metaDEGs). Many of the metaDEGs here identified encode for defense-related proteins (PR), TFs, and genes involved in plant defense hormone pathways, and were differentially expressed according to the duration and type of the stress imposed. As different *Arachis* species (*A. stenosperma* and *A. duranensis*) with contrasting levels of stress tolerance were used in this meta-analysis, we assume that the sets of genes identified (metaDEGs) are not genotype-dependent and are well-conserved in the genus, thus constituting interesting core genes involved in the high adaptability of these wild species to their native environments under stressful conditions.

As seen for different species^57–59^, the co-occurrence of two or more different stresses make the prediction of the genes that play a major role in this concerted defense response rather difficult, due to the existence of additive, subtractive and combinatorial effects and also the activation of specific genes and pathways that are triggered solely during stress combination^8^. This seems to be the case in *Arachis*, as our meta-analysis was able to predict only 7.2% of the genes observed in our de facto cross-stress transcriptome. This low predictability for genes responsive to combined stresses suggests the existence of dedicated pathways and genes that have evolved to mitigate the effects of stress combination, which, by being different, would not have been picked up by the meta-analysis. These findings also corroborate the need to conduct de facto combinatorial stress experiments to fully identify the genes exclusively prompted by simultaneous stresses.

Studies on hormonal signaling pathways and defense genes expression conducted in tomato and *A. thaliana*^13,14,30^ showed that not only the set of responsive genes between single and combined stresses are different, but they rely on different signaling hormonal pathways, which seem to interact and inhibit each other. Likewise, based on our DEGs functional categorization, allocation to different hormonal pathways, and drawing on our previous works^39,43,49^, we predicate that individual and combined abiotic and biotic stresses trigger different phytohormone pathways and hold different modulation and cross-talk points in *Arachis*. This assumption is reinforced in this study by the qRT-PCR expression analysis of marker genes from hormone signaling pathways which showed that whilst JA and ABA were the prevalent hormone pathways activated in response to RKN and drought stresses respectively, ET also appears as one of the main regulators in *Arachis* plants submitted to both stresses simultaneously.

Although the role of JA/ET in the defense network against necrotrophic pathogens has been described in different plant species^60^, the ET function as a regulator affecting both plant basal and systemic acquired resistance (SAR) in response to simultaneous stresses is less studied and seems to vary according to the plant-pathogen interaction^60–62^. Some reports suggest that stress-activated protein kinases (CDPKs, MAPKs) are responsible for integrating multiple environmental stresses via ET biosynthesis and stress perception^63^, while others point out that ET and JA cooperate through transcriptional induction of ERF (a regulator of ET signaling) during pathogen attack, whilst ABA might show an antagonistic effect on JA/ET pathogen defense signaling^30^ , or positively affect JA biosynthesis in the activation of defense responses^64^. The latter seems to be the case here, as the ET pathway marker ERF2 and the ET-responsive PDF1.2 genes were highly or exclusively induced in *Arachis* plants submitted to cross-stress treatment, in synchronism with the induction of JA-responsive LOX3 and VSP2 genes, whilst the ABA-dependent regulator bZIP and RD29B were only slightly induced or downregulated in this condition.

This protagonist role of the JA/ET pathways during *A. stenosperma* defense responses against *M. arenaria* observed here and in our previous studies^43,49^, has also been reported in sweet potato infected with *M. incognita*, in which the ET-dependent ERF and JA-dependent MYC TF genes were induced^65^, and in *Arabidopsis*^66^.

As plants need to balance the costs and benefits to initiate tolerance/resistance responses against a number of environmental constraints, they evolved a series of flexible signaling networks to optimize its adaptative responses. Cross-talk between these defense signaling hormonal pathways allows plants to tailor the appropriate defensive strategy to multiple stresses in a cost-effective manner^15^. In this direction, genes and regulators identified in this study which include those responsive to both biotic (RKN infection), abiotic (drought) and the combination of these stresses, constitute a valuable gene pool to provide new insights into the transcriptional regulatory networks trigged by isolated and combined stresses and help us to better understand the genetic sources controlling the natural ability of wild *Arachis* species to resist to a number of environmental challenges. Moreover, this core stress-responsive genes constitute precise targets for biotechnological purposes to develop multiple stress resistance in crops.

In this study, an endochitinase from *A. stenosperma* (*AsECHI*), was identified as one of the few genes commonly induced in response to drought, RKN infection, and combined stresses in the de facto cross-stress experiments. Although there are many studies using chitinases on the enhancement of plant resistance against fungi^67^, only a few reports on transgenic plants overexpressing chitinase genes to increase resistance against sedentary phytonematodes^68,69^ or tolerance to abiotic stresses, such as cold and drought^70,71^ have been conducted. To our knowledge, this is the first time that the overexpression of a plant endochitinase gene is proved to confer resistance to isolated and combined RKN infection and drought stresses.

This dual effect observed in plants overexpressing *AsECHI*, which increases tolerance to drought concomitant with a reduction of up to 30% in RKN infection, might be due to an increased accumulation of peroxidase activity in plants, as previously observed in transgenic tobacco^72^. In fact, peroxidases have a plural role in biotic and abiotic stress responses, as they act as signal molecules in the defense of plants against pathogen attacks and also contribute to increasing abiotic stress tolerance by counteracting the oxidative stress and also by favoring water retention in the cell walls, as suggested by^73^. Here, we demonstrated that transgenic *AsECHI*-OE plants exhibited higher RWC and reduced EL values than WT that suggest a greater ability for water retention and a low degree of membrane injury, with better protection against oxidative damage caused by isolated and combined stresses. In addition to peroxidase accumulation, the action of endochitinases can cause the liberation of glycoprotein oligomers in the plant cell wall or apoplast, which initiate defense signaling pathways such as those prompted by SA, also contributing to this increase in tolerance to biotic and abiotic stresses^72^. Therefore, the use of endochitinase genes such as *AsECHI* alone, or in combination with other plant defense genes, is an interesting sustainable strategy to improve crop tolerance and production.

With the onset of climate change, an increasing shift on the search for genes to be used for plant genetic engineering from individual to multiple stresses is occurring, as more sustainable crop management and disease control strategies are required. In the present study, we found common and specialized genes and pathways that coordinate plant responses to combined stresses in wild *Arachis*. The further understanding of the complex regulation of biotic and abiotic responses and their interactions in order to avoid *trade-off* effects that limit plant growth and yield will enable a more efficient biotechnological crop improvement.

## Methods

### Meta-analysis of *Arachis* RNA-Seq data

Previously published RNA-Seq data comprising 16 libraries of roots from *A. duranensis* and *A. stenosperma* plants submitted to abiotic and biotic stresses were used for the meta-analysis (Supplementary Table S1). A list of genes with an adjusted p-value (FDR) < 0.05 from each independently differential expression analyses (control × stressed) were used as input for the metaRNASeq R package^44^. RNA-Seq libraries from abiotic (dry-down and dehydration) and biotic (nematode inoculation) treatments (Supplementary Table S1) were analyzed separately. As the RNA-Seq libraries were obtained from different species, conditions, and protocols, we used the combined p-value with Fisher’s method for the two meta-analyses, as implemented in metaRNAseq R package as previously applied in other RNA-Seq studies^74–77^. This method allows the comparison of heterogenous datasets to find commonly regulated genes among all studies^78^. The genes with an adjusted p-value < 0.05 and the same expression trend (up or down regulated) for all the comparisons were considered as differentially expressed metaDEGs for Abiotic (A-metaDEGs) or Biotic (B-metaDEGs) stresses. The overlapping of A- and B-metaDEGs was conducted using the graphical representation of the common genes by the Eulerr on-line software (http://eulerr.co) and genes found in common between A- and B-metaDEGs were considered as core-metaDEGs, regardless of their expression trend (Supplementary Figure S3). The scripts used in this analysis are available in https://github.com/lbi-cenargen/MetaAnalysis/.

### Functional annotation and heatmaps

Genes identified as DEGs were functionally annotated by Mercator^79^, using default parameters and used as input for the boxplot in the ggplot2 package in R^80^. Visualization of commonly expressed genes among the 16 RNA-Seq libraries was conducted by InteractiVenn (http://www.interactivenn.net/). Heatmaps showing the expression profile of DEGs were made in gplots package^81^ using average expression values for genes in the biotic and abiotic treatments^11^. For dotplot, we used the expression values from the core-metaDEG and crossDEG as input for ggplot package^80^.

### *Arachis stenosperma* cross-stress assays and library sequencing

#### Plant materials and bioassays

Six-week-old *A. stenosperma* (accession V10309) plants grown at 70% of field capacity (FC) under greenhouse conditions were submitted to four treatments (well-irrigated control, cross-stress, nematode infection and drought), with each treatment comprising three sets (biological replicates) of 20 plants. At the end of treatments, plant roots were collected in liquid nitrogen and stored at − 80 ºC for RNA extraction.

For nematode infection, *M. arenaria* (race 1) juveniles (J2) were extracted from tomato roots as previously described^40^. Plants were inoculated with 10,000 J2 and kept well-watered under greenhouse conditions for 7 days after inoculation (DAI). The susceptible peanut cultivar (*A. hypogaea* cv. Runner) was also inoculated as a positive control.

For the dry-down assay, plants were submitted to a gradual decrease of soil moisture (dry-down) by the withholding of irrigation for 7 days until 25% FC as described before^82^.

For the cross-stress assay, plants were submitted to combined *M. arenaria* inoculation and drought stresses in greenhouse conditions, as six-week-old plants kept at 70% FC were inoculated with 10,000 J2 of *M. arenaria* and submitted to water withdrawal for seven days. At the 7th day plants were collected and stored as above.

#### Cross-stress RNA-Seq sequencing and analysis

Total RNA was extracted from roots of the three biological replicates from each treatment (cross-stress, nematode, drought, and control) using a modified lithium chloride protocol^83^ and further purified with Invisorb Plant RNA Mini Kit (Invitek, Berlin, Germany). RNA was reverse transcribed and analyzed according to Morgante et al., (2013). In total, 12 paired-end cDNA libraries were constructed using the TruSeq™ Stranded Total RNA LT Sample Prep Kit from Illumima (Illumina Inc., San Diego, CA, USA) and sequenced by Macrogen (Seoul, Korea) using HiSeq-4000, 150 bp (Illumina Inc., San Diego, CA, USA). Illumina raw reads were analyzed and quantified as before^43^ (PRJNA284674).

### AsECHI overexpression in *Arabidopsis thaliana*

The complete coding sequence (795 bp) of an endochitinase-encoding gene from *A. stenosperma* (*AsECHI*) was synthesized and cloned under the control of the *A. thaliana* actin 2 promoter in the binary vector pPZP_BAR by Epoch Life Science Inc. (Sugar Land, TX, USA). pPZP-BAR is derived from pPZP_201BK_EGFP^85^ in which we replaced the hygromycin resistance gene by the bar gene from *Streptomyces hygroscopicus*^86^ for glufosinate ammonium herbicide resistance. Wild-type (WT) *A. thaliana* plants (ecotype Col-0) were transformed using the pPZP-AsECHI vector as described by^87^. The eGFP-positive and glufosinate-resistant transformants were grown in a controlled growth chamber (21 °C, 12 h photoperiod and light intensity of 120 μmols m^−2^ s^−1^) to obtain transgenic *AsECHI* overexpressing (OE) lines, as described by^88^. All biotic and abiotic stress treatments were conducted using homozygous *AsECHI*-OE lines at the T2 generation.

#### qRT-PCR analysis

The expression levels of *AsECHI* transgene in *A. thaliana* OE lines and WT plants and of the hormone pathways marker genes in *A. stenosperma* were analyzed by qRT-PCR, using total RNA extracted with the RNeasy Plant Mini Kit (Qiagen, Hilden, Germany) according to^84^. qRT-PCR reactions were performed in three biological replicates on the StepOne Plus Real-Time PCR System (Applied Biosystems, Foster City, CA, USA) and the relative quantification (RQ) of mRNA levels estimated using the SATqPCR web tool^89^. The specific primers of the target and reference genes are described in the Supplementary Table S7.

#### AsECHI-OE lines assays

Four-weeks-old *A. thaliana* plants grown on sand:substrate mixture (2:1; v:v) were submitted to isolated and coupled biotic and abiotic stress assays. For the nematode assays, ten plants were inoculated with *M. incognita* J2 (1,000) as described by Morgante et al. (2013) and kept at the growth chamber conditions. At 60 DAI, the number of nematode females on roots was assessed and statistically compared using the t-test (p < 0.05)^88^.

For the dry-down assay, irrigation of 20 plants was suspended for nine days (when plants showed wilting symptoms) in the stressed group, whilst the control group of individuals was kept under 70% FC. At the end of the treatment, three leaf discs (0.4 cm^2^) were collected per individual for the determination of the Relative Water Content (RWC) and electrolyte leakage (EE), according to Vinson et al. (2020)^90^.

For the cross-stress assay, nine plants per OE line and WT were first submitted to water withdrawal for seven days and then inoculated with *M. incognita*, as described above. Plants were maintained under this combined stress condition (drought and nematode) for four additional days, after which all plants were rewatered. The non-inoculated plants were maintained at 70% FC. The number of *M. incognita* females in the OE lines was assessed at 60 DAI as described above.

## Supplementary Information

Supplementary Information.

Supplementary Table S2.

Supplementary Table S3.

Supplementary Table S4.

Supplementary Table S6.
