# Supplementary material for: Defining the combined stress response in wild Arachis
Source: Sci Rep. 2021 May 27;11:11097. doi: 10.1038/s41598-021-90607-7 (PMC8160017; doi:10.1038/s41598-021-90607-7)
Supplement: Supplementary file 1 — Supplementary Information. [file 41598_2021_90607_MOESM1_ESM.pdf]

Supplementary Files

Defining the combined stress response in wild *Arachis*

Ana Paula Zotta Mota[a,b,c,1,2], Ana Cristina Miranda Brasileiro[a,c], Bruna Vidigal[a,c], Thais Nicolini Oliveira[a,c], Andressa da Cunha Quintana Martins[a,c], Mario Alfredo Passos Saraiva[a,c], Ana Claudia Guerra de Araújo[a,c], Roberto C. Togawa[a,c], Maria Fatima Grossi-de-Sá[a,c,d], Patricia Messenberg Guimaraes[a,c]

Supplementary Figure S1: Relative quantification of *AsECHI* expression in four *A. thaliana* OE lines using *AtACT* and *AtEF1α* reference genes as normalizers.

Supplementary Figure S2: Dry-down and nematode assays of four *A. thaliana AsECHI*-OE lines (OE1, OE2, OE6 and OE7) and WT plants. a) Control (CTR) and drought stressed plants (STR) submitted to dry-down assay in the first and last (9th) days of the experiment. Relative Water Content (RWC) and electrolytic leakage (EE) percentage at the 9th day of drought imposition. b) Average number of *M. incognita* females per gram of *A. thaliana* root. Values are mean of 15 individuals and significant differences between WT and OE lines are marked with an asterisk.

Supplementary Figure S3: Flowchart of the analyses used for meta-analysis. *A. stenosperma* inoculated with nematode at 3DAI, 6DAI and 9DAI (SN3, SN6, SN9), *A. stenosperma* submitted to dehydration (SDHY) and dry-down (SDD). *A. duranensis* inoculated with nematode at 3DAI, 6DAI and 9DAI (DN3, DN6, DN9), *A. duranensis* submitted to dehydration (DDHY) and dry-down (DDD).

Supplementary Table S1: *Arachis* spp. libraries (RNA-Seq) used in the meta-analysis and their NCBI accession numbers.

Supplementary Table S2: A-metaDEGs and the expression values in both *A. stenosperma* and *A. duranensis*, for both dry-down and dehydration assays. DDHY: *A. duranensis* submitted to dehydration, SDHY: *A. stenosperma* submitted to dehydration, DDD: *A. duranensis* submitted to dry-down, SDD: *A. stenosperma* submitted to dry-down.

Supplementary Table S3: B-metaDEGs and the expression values in both *A. stenosperma* and *A. duranensis*, in 3 days after inoculation (SN3 and DN3), 6 days after inoculation (SN6 and DN6) and 9 days after inoculation (SN9 and DN9).

Supplementary Table S4: crossDEGs of *A. stenosperma* and their expression values in Log2FC.

Supplementary Table S5: Illumina HiSeq 4000 sequencing data from *A. stenosperma* quantified in the *A. duranensis* reference genome.

Supplementary Table S6: Commonly regulated genes between core-metaDEGs and *de facto* combined stress libraries.

Supplementary Table S7: Primer sequences used for qRT-PCR analysis in this study.

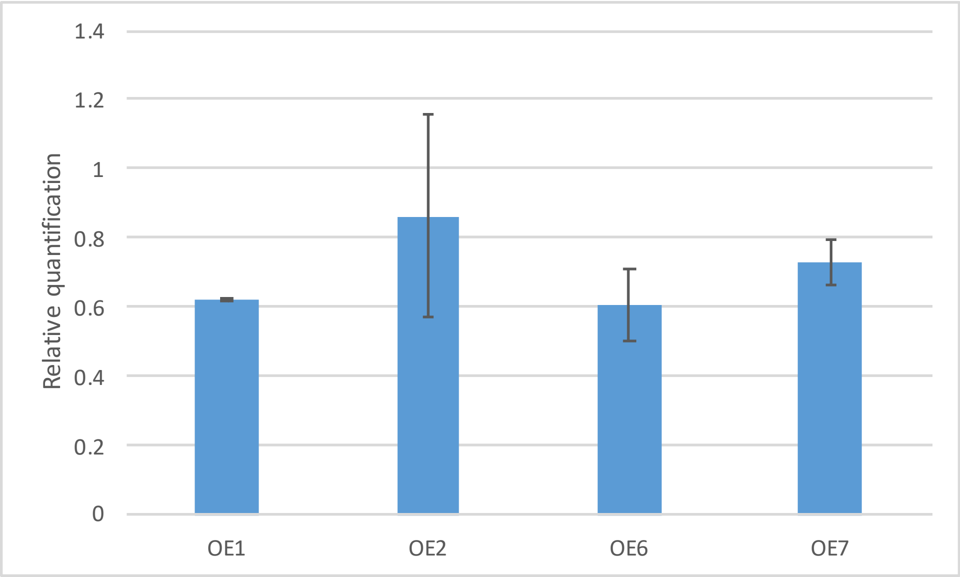

Supplementary Figure S1: Relative quantification of *AsECHI* expression in four *A. thaliana* OE lines using *AtACT* and *AtEF1α* reference genes as normalizers.

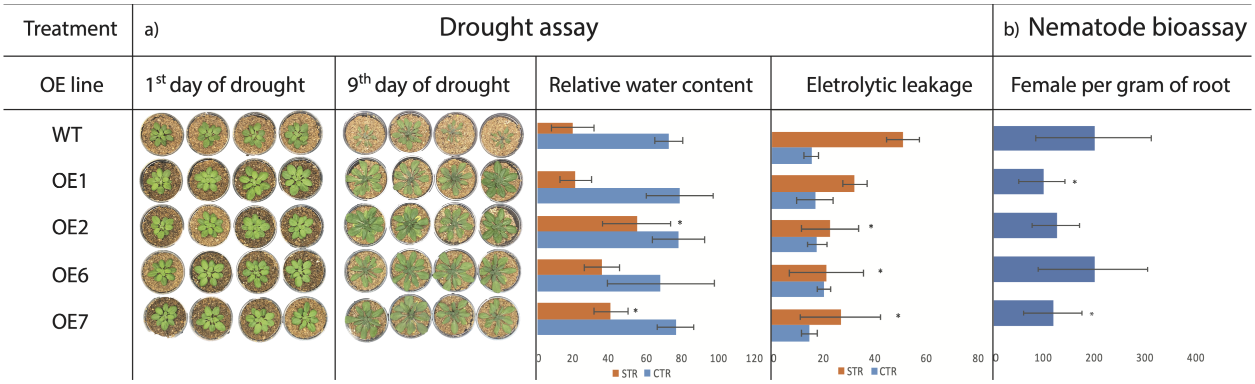

Supplementary Figure S2: Dry-down and nematode assays of four *A. thaliana AsECHI*-OE lines (OE1, OE2, OE6 and OE7) and WT plants. a) Control (CTR) and drought stressed plants (STR) submitted to dry-down assay in the first and last (9th) days of the experiment. Relative Water Content (RWC) and electrolytic leakage (EE) percentage at the 9th day of drought imposition. b) Average number of *M. incognita* females per gram of *A. thaliana* root. Values are mean of 15 individuals and significant differences between WT and OE lines are marked with an asterisk.

Supplementary Figure S3: Flowchart of the analyses used for meta-analysis. *A. stenosperma* inoculated with nematode at 3DAI, 6DAI and 9DAI (SN3, SN6, SN9), *A. stenosperma* submitted to dehydration (SDHY) and dry-down (SDD). *A. duranensis* inoculated with nematode at 3DAI, 6DAI and 9DAI (DN3, DN6, DN9), *A. duranensis* submitted to dehydration (DDHY) and dry-down (DDD).

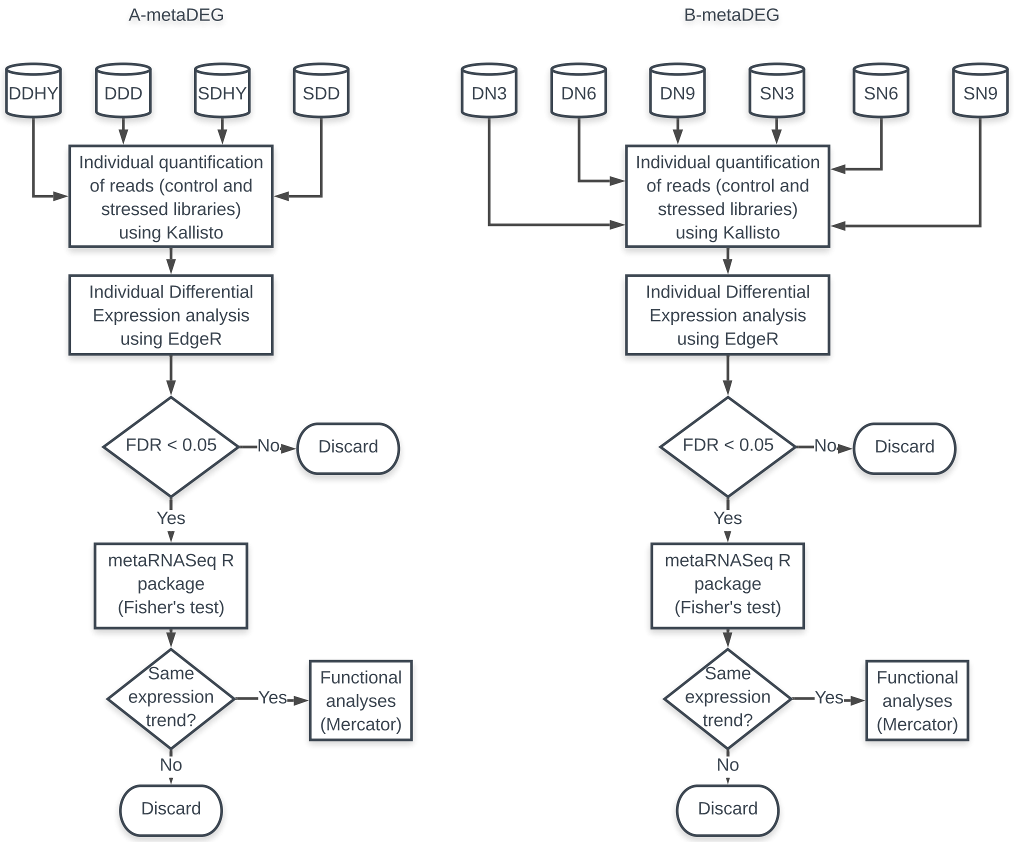

Supplementary Table S1: *Arachis* spp. libraries (RNA-Seq) used in the meta-analysis and their NCBI accession numbers.

Accession number	Specie	Stress	Tissue	Time-point	Total reads	Reference
SAMN03734821	*Arachis stenosperma*	*Meloidogyne arenaria* inoculation	Roots	0 DAI (control)	6,651,071	Guimaraes et al., 2015
SAMN03734822	*Arachis stenosperma*	*Meloidogyne arenaria* inoculation	Roots	3 DAI	7,162,500	Guimaraes et al., 2015
SAMN03734823	*Arachis stenosperma*	*Meloidogyne arenaria* inoculation	Roots	6 DAI	6,333,182	Guimaraes et al., 2015
SAMN03734824	*Arachis stenosperma*	*Meloidogyne arenaria* inoculation	Roots	9 DAI	6,202,969	Guimaraes et al., 2015
SAMN08225801	*Arachis stenosperma*	Drought (Dehydration)	Roots	0 minutes (control)	12,219,081	Vinson et al., 2018
SAMN08225802	*Arachis stenosperma*	Drought (Dehydration)	Roots	25 - 150 minutes	12,587,737	Vinson et al., 2018
To be submitted	*Arachis stenosperma*	Drought (Dry-down)	Roots	0 DAI (control)	100,326,990	This study
To be submitted	*Arachis stenosperma*	Drought (Dry-down)	Roots	7 DAI	112,787,062	This study
SAMN08225796	*Arachis duranensis*	*Meloidogyne arenaria* inoculation	Roots	0 DAI (control)	66,932,681	Mota et al., 2018
SAMN08225797	*Arachis duranensis*	*Meloidogyne arenaria* inoculation	Roots	3 DAI	65,043,113	Mota et al., 2018
SAMN08225798	*Arachis duranensis*	*Meloidogyne arenaria* inoculation	Roots	6 DAI	64,288,593	Mota et al., 2018
SAMN08225799	*Arachis duranensis*	*Meloidogyne arenaria* inoculation	Roots	9 DAI	60,877,309	Mota et al., 2018
SAMN08225788	*Arachis duranensis*	Drought (Dehydration)	Roots	0 minutes (control)	9,085,802	Vinson et al., 2018
SAMN08225800	*Arachis duranensis*	Drought (Dehydration)	Roots	25 - 150 minutes	8,453,190	Vinson et al., 2018
To be submitted	*Arachis duranensis*	Drought (Dry-down)	Roots	0 DAI (control)	134,098,748	This study
To be submitted	*Arachis duranensis*	Drought (Dry-down)	Roots	7 DAI	123,371,298	This study

Supplementary Table S5: Illumina HiSeq 4000 sequencing data from *A. stenosperma* quantified in the *A. duranensis* reference genome.

		**Nematode**	**Drought**	**Combined**	**Control**
Replicate 1	Number of raw reads	34,173,582	35,004,558	37,568,042	29,324,662
	Number of reads after trimming	30,028,088	31,823,728	33,512,716	26,572,592
	Number of quantified reads into the reference	10,631,810	9,189,171	10,385,619	8,469,816
	Total number of gene models	25,446	24,848	25,386	25,943
Replicate 2	Number of raw reads	34,236,660	37,275,858	33,190,158	36,116,360
	Number of reads after trimming	31,699,592	33,061,206	30,115,720	31,430,734
	Number of quantified reads into the reference	11,653,431	9,679,977	8,651,041	9,593,936
	Total number of gene models	25,715	25,266	25,227	24,825
Replicate 3	Number of raw reads	31,200,332	40,506,646	36,428,454	34,885,968
	Number of reads after trimming	29,136,428	37,119,422	31,889,266	30,148,320
	Number of quantified reads into the reference	10,650,358	10,935,791	9,641,333	8,735,584
	Total number of gene models	25,330	25,695	24,986	24,998
DEGs	Number of genes with FDR < 0.05	1,251	6,102	3,345	N/A

Supplementary Table S7: Primer sequences used for qRT-PCR analysis in this study.

Primer name	Species target	Description	Sequence 5' 3'	Reference
AtACT2	*A. thaliana*	Reference gene Actin 2 of A. thaliana	CTTGCACCAAGCAGCATGAA	Czechowski et al. 2005
			CCGATCCAGACACTGTACTTCCTT	
AtEF-1α	*A. thaliana*	Reference gene Elongation Factor1 of A. thaliana	TGAGCACGCTCTTCTTGCTTTCA	Czechowski et al. 2005
			GGTGGTGGCATCCATCTTGTTACA	
AsECHI (Cht2)	*A. thaliana*	*A. stenosperma* endochitinase	GCCCATGGCTTCTACACCTA	Carmo et al. 2019
			CCCCATTCAAATTCATCTGG	
60S	*A. stenosperma*	Reference gene 60S of *A. stenosperma*	CAACAACGGAGACATCAACG	Morgante et al., 2011
			ATCACTGCCACCCAGAAAAC	
GAPDH	*A. stenosperma*	Reference gene GAPDH of *A. stenosperma*	TGGAGTGAGAGGTGCATTTG	Morgante et al., 2011
			TCTTTTGACGACCAGGGAAC	
AsERF2	*A. stenosperma*	Markers gene from ERF2 pathway of A. stenosperma	AGAGGAGGGGCCAAATAAGA	This study
			CAGCAGTGTCAAAGGTTCCA	
AsbZIP	*A. stenosperma*	Marker gene from bZIP pathway of A. stenosperma	TGATGCAGCAGTTCCTGTTC	This study
			GCTGCACATTTTCGTCTGAA	
AsVSP2	*A. stenosperma*	Marker gene from VSP2 pathway of A. stenosperma	AGCACCTCCACTTCCAGAGA	This study
			GGACTGTGGCCTTCCAGTTA	
AsRD29B	*A. stenosperma*	Marker gene from RD29B pathway of A. stenosperma	GAACGAGACTTGCCAGAGCT	This study
			TGTGGCTGCTGGTACTTGAG	
AsPDF1.2	*A. stenosperma*	Marker gene from PDF1.1 pathway of A. stenosperma	TGCGACGACCACTGCAAGAACA	This study
			TTTCTGGTGCACCAACAGCGGA	
AsLOX3	*A. stenosperma*	Marker gene from LOX3 pathway of A. stenosperma	AGAAAGACGAGCCATGGTGG	This study
			TAGGATGAAACCGCCGTACG	
